# From Vulnerability to Resilience: Roles of Community Support for Well-Being of Older Rohingya Refugees in Bangladesh

**DOI:** 10.1155/jare/4538913

**Published:** 2025-11-24

**Authors:** Mohammed Mamun Rashid, Md. Anamul Hoque, Sharmin Akter Joya

**Affiliations:** ^1^Department of Media Studies and Journalism, University of Liberal Arts Bangladesh (ULAB), Dhaka, Bangladesh; ^2^Sesame Workshop, Dhaka, Bangladesh; ^3^Department of Criminology and Police Science, University of Chittagong, Chittagong, Bangladesh

**Keywords:** aging, community support, elder care, forced displacement, refugee health, Rohingya

## Abstract

The Rohingya people have been subjected to egregious human rights abuses, culminating in a mass exodus to the Cox's Bazar district of Bangladesh in 2017. This study investigated the knowledge, attitudes, and practices surrounding older persons within the Rohingya refugee community while also elucidating the present community support mechanisms crucial for their well-being, through a sequential mixed-methods approach. The study specifically focuses on older Rohingya persons aged 60 years and above. A printed survey questionnaire was administered to 377 Rohingya refugees residing in camps, complemented by key informant interviews (KIIs) conducted with eight experts and community leaders to gain nuanced insights into the challenges and support systems. The quantitative data were analyzed using descriptive statistics to determine the prevalence of specific knowledges, attitudes, and practices while qualitative data from KIIs were subjected to thematic analysis in alignment with numerical facts, identifying recurring themes related to community support and vulnerabilities. The findings revealed that a significant majority (89.92%) of older Rohingya refugees lived within extended family structures, underscoring the importance of intergenerational coresidence, and a substantial proportion of respondents (87.23%) acknowledged and respected the wisdom and experience of elders. Family bonds, sociocultural, and religious factors played significant roles in ensuring personal care. However, older persons faced numerous vulnerabilities related to insufficient food, unfriendly infrastructure, inadequate healthcare, poor social services, and so on. This paper concludes with several recommendations particularly to strengthen community support for the well-being of older Rohingya refugees living in Bangladesh.

## 1. Introduction

Population aging is a global and irreversible trend, driven by demographic transitions characterized by longer lifespans and smaller family sizes, a pattern observable even in countries with younger populations [1]. The number of people aged 65 and older is projected to more than double from 761 million in 2021 to 1.6 billion by 2050, with those aged 80 and above growing even faster (Department of Economic and Social Affairs [2]). The global refugee crises have displaced millions, with many older refugees experiencing challenges due to their distinct needs, for instance, movement to far away from the place of origin, alone living, lack of family care, difficulties in accessing healthcare services, etc. Amid this global aging phenomenon, the forced displacement of the Rohingya population from Myanmar to Bangladesh has resulted in one of the most pressing humanitarian crises, with over 954,131 stateless refugees living in 33 overcrowded camps as of July 2024, in Cox's Bazar district. Older refugees, constituting 17,117 females and 22,280 males aged 60 and above, face heightened vulnerabilities due to poor living conditions, inadequate access to age-appropriate services, and social isolation [3].

Despite the above challenges, the specific needs of older Rohingya refugees, including limited mobility, heightened health risks, and psychological stress, have been largely neglected in humanitarian responses [4]. Conditions in the camps are perilous, with temporary shelters lacking proper ventilation and exposing residents to extreme heat, fire hazards, and frequent arson incidents [5]. Rohingya people continue to endure a severe human rights crisis within these camps, exacerbating their already dire living conditions [6]. Humanitarian efforts involving more than 117 partners, including 10 UN agencies and numerous NGOs, have prioritized sectors such as education, food security, health, livelihoods, nutrition, protection, shelter, and water, sanitation, and hygiene (WASH), with cross-cutting areas such as age and disability inclusion, accountability to affected people, and emergency preparedness [7].

Community engagement has been identified as a critical factor in enhancing the well-being and resilience of vulnerable populations in resource-constrained environments, where formal support systems are limited [1, 8]. Unfortunately, the specific plight of older refugees, particularly in fostering resilience through community support, remains underexplored [9]. This study aims to investigate the intersection of vulnerability and resilience among older Rohingya refugees by focusing on the role of community support in improving their well-being. Specifically, it seeks to examine the knowledge, attitudes, and practices (KAP) within the Rohingya community concerning their older members and to explore strategies and interventions for enhancing community engagement.

## 2. Literature Review

The concept of general well-being is described as a holistic state of physical, emotional, social, and mental health that reflects an individual's overall quality of life [10]. Longo et al. [11] argued that well-being encompasses both objective measures, such as access to basic resources, and subjective experiences of life satisfaction. For the older, well-being extends beyond survival to include autonomy, social engagement, and mental health [12]. Hachem et al. [13] also claimed that the well-being of refugees has been extensively studied, with a predominant focus on younger and adult populations, leaving the challenges faced by older refugees largely overlooked. Older refugees face even greater challenges, as displacement exacerbates vulnerabilities [14]. Chowdhory [15] described the concept of “dual vulnerability” where the physical challenges of aging are compounded by displacement. Refugee camps often lack adequate food, sanitation, and healthcare, neglecting older individuals' needs [16]. For older Rohingya refugees, these difficulties are worsened by prolonged displacement and statelessness [17]. Nasar et al. [18] highlighted these vulnerabilities, noting that older Rohingya refugees are often deprived of basic necessities. Despite growing studies on refugee well-being since 2019, Khan et al. [19] pointed to a lack of research on the specific challenges faced by older refugees, particularly in accessing essential services. Existing studies have filled this gap by discovering barriers faced by older Rohingya refugees.

It is important to note that when refugee vulnerability is addressed in the literature, it overwhelmingly centers on women and children [20]. While these groups undoubtedly face significant hardships, the needs of older refugees—particularly in terms of health, mobility, and emotional well-being—remain substantially underexplored [21]. This gap is especially evident in humanitarian research concerning the Rohingya population, where programming and policy responses have historically prioritized maternal health, child nutrition, and protection for women and girls [22]. As a result, older persons are often rendered invisible in academic discourse and field-based interventions [23]. The current study seeks to address this imbalance by contributing novel data and analysis focused on older Rohingya refugees.

Special needs of older refugees, including access to specialized healthcare, mental health services, and social support, have been inadequately addressed. In countries such as Pakistan, Uganda, and Bangladesh, older refugees face heightened risks, exacerbated by inadequate nutrition and healthcare access [24]. Similarly, studies in Sudan, Ethiopia, and Russia focused on broader refugee issues, overlooking the multiple vulnerabilities of older refugees [25, 26]. The present study efforts to fulfill this gap by focusing on the physical and mental well-being of older Rohingya refugees in Bangladesh, advocating for more inclusive and age-sensitive humanitarian interventions across refugee camps.

Community support, both formal and informal, is crucial for the well-being of refugees, especially the older, by addressing their specific vulnerabilities and fostering resilience [27]. Key figures such as camp spokesmen, religious leaders, health workers, and informal actors such as youth volunteers and cultural activists are essential in building support networks [28, 29]. Strang and Quinn [30] specified that structures of refugee camps often prioritize children and women, but older individuals are underserved. In camps across Bangladesh, Pakistan, Kenya, and Afghanistan, while there are health posts and counseling centers, they often fail to meet the specialized needs of older refugees. Kabue et al. [31] also instance that community efforts focus on refugee women and children in Kenya, with little regard for the older. Similarly, in Pakistan, initiatives are geared toward food security and youth, neglecting older care [32]. Afghanistan's programs emphasize youth and food security but overlook the older need for specialized care and psychological support [33–35]. Rohingya camps in Bangladesh also fall short, with a focus on general health rather than the intensive support needed for older refugees [36].

Community services, a key sector in the international aid response led by UNHCR, originally focused on social services and providing care for refugees who could not meet their needs through basic camp provisions. Over the past decade, its scope has expanded significantly, aligning with UNHCR's shift toward a community development approach. Despite this expansion, community services remain underfunded and less influential compared to essential sectors such as food, health, water, and sanitation. A recent UNHCR evaluation highlighted that, although community services are crucial for addressing the physical and psychological needs of vulnerable refugees, they receive significantly less attention [37]. Therefore, this study has an innovative contribution by highlighting the emerging needs to increase community engagement strategies and interventions that specifically cater to older Rohingya refugees, advocating for a more inclusive and targeted approach.

## 3. Aims of the Study

While extensive research has explored refugee populations' general well-being, a significant gap exists in understanding support mechanisms for older refugees, particularly regarding community engagement and attitudes toward their care. The existing literature predominantly focuses on younger refugees, leaving older individuals' specific needs underexamined [38, 39]. This study addresses these gaps by investigating the relationship between community support, vulnerability, and resilience among older Rohingya refugees in Bangladeshi camps. The aims of this study are as follows:

1. To assess the current levels of KAP related to older individuals' well-being in Rohingya refugee camps.
2. To explore strategies and interventions promoting effective community engagement to enhance older refugees' well-being.
3. To examine community support's impact on older refugees' vulnerability and resilience in resource-constrained environments.
4. To provide evidence-based recommendations for minimizing vulnerability and bolstering resilience among older refugees in Bangladeshi Rohingya camps.

By addressing these aims, this study seeks to contribute to understanding community-based support systems for older refugees and inform policy and practice in humanitarian settings.

## 4. Methodology and Methods

### 4.1. Study Design

Study design is the framework that guides the achievement of objectives. According to Akanda [40], it serves as the overall structure of research, enabling the delivery of evidence required to address the research problem. It involves a step-by-step process that includes selecting the appropriate methods, determining the sampling strategy, collecting data, ensuring reliability and validity, and conducting analysis to meet the objectives. In this particular study, a quantitative approach was predominantly employed for data collection. A questionnaire survey was utilized for this purpose. Additionally, Creswell [41] highlights that a qualitative method can complement quantitative research by providing explanations for the relationships in a causal model. Consequently, the authors incorporated the key informant interview (KII) technique to gather opinions from community members and professional experts. This study employed a sequential mixed-methods design, in which quantitative and qualitative data were collected in two consecutive phases. The household survey provided descriptive and numerical data on the conditions of older Rohingya persons, while the KIIs offered interpretive insights that explained and contextualized those quantitative findings. This sequential approach ensured that qualitative evidence complemented quantitative patterns, allowing for a deeper understanding of both the structural and experiential dimensions of vulnerability and resilience among older refugees [42, 43].

### 4.2. Study Location and Respondents

Rohingya camps were the main area for conducting the household survey. Respondents from 15 camps (Camps 1E, 1W, 3, 4, 5, 6, 8East, 11, 12, 13, 14, 16, 18, 19, and 20Ext) contributed to the survey. Bhardwaj [44] suggests that random sampling is unbiased and ensures that each unit has an equal probability of inclusion. However, convenience sampling is more appropriate in challenging areas and is easy to implement for large-scale data collection. It is also cost-effective. A useful webpage, Raosoft [45], provides a sample size calculator that suggests taking a sample of 377 units if the population number is unknown. Due to resource limitations, a total of 377 respondents (131 females and 246 males) were approached for the survey. They provided information about 377 older persons (171 females and 206 males) and issues and concerns indicated in the questionnaire. In this study, the term *“older person”* refers to individuals aged 60 years and above, following the definition adopted by the World Health Organization [46] and the Bangladesh National Policy on Older Persons [47]. This age threshold aligns with international and national standards on aging and allows the study to maintain consistency with comparable research on refugee populations in Bangladesh and South Asia. While the primary data were collected from household members rather than older individuals themselves, this approach was chosen due to ethical and logistical barriers—particularly language constraints, limited mobility, and the protection of vulnerable populations during fieldwork. The major inclusion criteria for respondents of the household survey were being at least 18 years old, living with an older person, willing to provide information, living in a refugee camp, and good cognitive capacity. The study therefore draws upon proxy responses from caregivers and family members, complemented by insights from professionals and community leaders engaged with older persons' welfare. The authors used a combination of closed-ended and open-ended questions, comprising 31 questions aligned with the study objectives. This methodological choice is acknowledged as a limitation but provides an ethically sound foundation for exploring broader community perceptions [21].

KII are advantageous for their versatility and flexibility [48]. Experienced and knowledgeable participants about research topic is significantly helpful to obtain more accurate data. Dery et al. [49] also revealed that interviews are reciprocal and helpful in exploring experiences and interpretations from informants, allowing for follow-up questions. Muellmann et al. [50] recommend that 6–12 key informants are sufficient for a specific and narrowed-down topic. Having more key informants may alter and confound results. For this study, Eight KIIs participants (five Rohingya and three Bangladeshi) were conducted with a purposively selected group of community leaders, NGO workers, religious leaders, and humanitarian professionals based on their work in humanitarian organizations and at least 3 years of experience, particularly in relation to the welfare of older Rohingya people (Table 1). This qualitative component was designed to complement the survey findings by capturing expert and contextual perspectives. Although KIIs provided valuable insights, the authors acknowledge that future research would benefit from direct participatory interviews or focus groups with older refugees to amplify their lived experiences.

### 4.3. Data Collection

The authors conducted fieldwork from October 2024 to January 2025. The author visited several houses and interviewed household members using a printed questionnaire. Two social workers, one female and one male, assisted in this process. The questionnaire covered biographic information, household location, knowledge relating to nutritious foods, mental health, social activities, health services, home-based care, decision-making of older persons; discrimination, age-friendly space, organizational supports, recreation, dignity, utilization of wisdom and experience gathered by older persons; and major barriers to access to health facilities, effective mechanisms for disseminating health service messages regarding older persons in wider communities, and more.

KII were conducted using handwritten notes as the primary data collection tool. A KII protocol was prepared, focusing on key findings of the KAP survey. It was a sensitization tool to reflect the actual scenario among KII participants. It also addressed questions such as awareness building, family care, emotions, health services, age-friendly provisions, and message dissemination by strengthening community supports. With the consent of the participants, the author ensured that handwritten notes were taken during the discussions.

### 4.4. Reliability and Validity of Data

Sarstedt et al. [51] and Halim et al. [52] strongly agreed that data reliability and validity are prerequisites for high-quality research. The data collector asked the same question two or three times from different perspectives to ensure consistent answers. Two social workers, one female and one male, assisted the author in verifying the data's validity by cross-checking the respondents' answers. When applicable, the data were also verified with family members.

Cypress [53] described qualitative research as a process-oriented approach where the expectation of data reliability and validity is minimal during postevaluation. The author facilitated each KII by following a step-by-step procedure with a data collection protocol. For example, this included setting inclusion criteria for participants, selecting knowledgeable and experienced community people and professionals on the topic of older persons' vulnerability and resilience, managing time properly (with each interview taking approximately half an hour), using subquestions to elicit accurate answers, and ensuring that handwritten notes are taken. Quantitative facts and KII notes were triangulated by using original data sources to explore the research objectives.

### 4.5. Data Analysis Procedure

The data from the printed questionnaire were entered into the Statistical Package for the Social Sciences (SPSS) software. The data were then analyzed and organized sequentially according to the objectives of the study. This included respondents' profiles and themes. Major themes are affiliation with family members, recognition and discrimination, healthcare services, organizational provisions, age-friendly spaces, home-based supports and devices, and information dissemination. The methodological framework of this study combines quantitative and qualitative strands through triangulation, ensuring validity and coherence. Quantitative data established statistical associations, while qualitative narratives illuminated the sociocultural meanings behind these patterns. Following the integration principles outlined by Fetters et al. [54] and Noble & Heale [55], this design strengthened credibility through convergence, complementarity, and expansion of data sources. Both data streams were interpreted together in the Discussion section to highlight intersections between vulnerability, resilience, and community support mechanisms. The ATLAS.ti software (Version 8) was utilized for coding and categorizing the qualitative data. The findings section of the study was structured based on both quantitative and qualitative analyses. Similar types of codes and homogenous title of survey data were considered to emerge themes. Emerged themes had matched with research questions. Based on data, the main theme, i.e., vulnerability to the resilience of older refugees through community support, was divided into subthemes, and finally, the subthemes were clustered into different categories to answer the research questions more specifically.

### 4.6. Ethical Considerations

This study was conducted in accordance with internationally recognized ethical principles, including the Declaration of Helsinki and the Belmont Report. While formal approval from an institutional review board was not obtained, all procedures were designed and implemented to uphold high ethical standards throughout the research process. Participants in both the household survey and KIIs were fully informed about the academic nature of the research. Participation was voluntary, and respondents were assured of their right to decline or withdraw without consequence. For illiterate participants, the consent form was read aloud in their native language, and verbal consent was obtained in the presence of an independent witness. To ensure confidentiality and anonymity, no personally identifiable information was collected. All data were securely stored with restricted access, and only the authors and two supporting social workers were aware of the identities of participants. Anonymity of key informants was preserved using coded identifiers in all transcripts and publications. Special care was taken in the wording and delivery of questions to ensure cultural appropriateness and to preserve the dignity of the Rohingya population. Given the inclusion of vulnerable groups—particularly older persons and women—interviews were conducted in private and safe settings, and gender-matched interviewers were employed where necessary. All data collectors received training in ethical fieldwork, including informed consent, confidentiality, and culturally sensitive interviewing. These procedures ensured the protection of participants' rights and well-being and maintained the integrity of the research process.

## 5. Findings

### 5.1. Respondents' Profile

A total of 377 respondents (131 females and 246 males) provided data about 377 older persons. The age, marital status, education, occupation, and family size of the respondents were briefly described.

### 5.2. Age

Overall, the majority of respondents fell within the age group of 31–40 years (29.97%), followed by 41–50 (26.80%). A total of 10.34% of respondents were from 51 to 60 years, and 14.85% were more than 60. However, 14.33% of the total respondents were 21–30, while 3.71% were from 18 to 20 years.

### 5.3. Marital Status

Among respondents, 5.96% were unmarried, while 84.28% were married. A total of 7.86% were widowed, and 1.90% were divorced.

### 5.4. Education

Among respondents, 59.95% were illiterate, and 30.50% had education from Grade 2 to 5, followed by 7.69% with grade 6 to 10. Notably, 1.86% of total respondents had education beyond Grade 10.

### 5.5. Occupation

All refugee respondents arrived in 2017. This study recorded their occupations in Myanmar. Among females, 81.68% were homemakers, followed by 16.12% in day labor and agricultural activities, and 2.20% were students. Among males, 51.94% were employed in farming (crop/fisheries/cattle) and day labor, followed by 28.71% in small business. 13.61% of them were in private jobs, and 5.74% were students.

### 5.6. Family Size

The family size of selected households was 5.38. Among family members, girls constituted 19.34%, boys constituted 19.21%, females constituted 31.21%, and males constituted 30.24%.

### 5.7. KII Participants

A total of five Rohingya and three host community members were approached for interviews. The age range of the majority of respondents (62.5%) was between 18 and 35 years. All respondents were married. They had diverse occupations and resided in different camps and locations within the host community.

### 5.8. Affiliation With Family Members

Among 377 older persons, 147 females and 192 males lived in joint families. The rest of the 24 females and 14 males lived with only their spouses, alone, or with other relatives. 87.23% of total respondents believed that the experiences of the older were helpful for decision-making in the family. The rest (12.77%) had a negative response or were unaware of this. A total of 79.58% of respondents realized that keeping extra nutritious foods for the older was important. Additionally, 81.70% of total respondents acknowledged that living with family was beneficial for the mental health of an older person. KII Participant 1 stated the following:

“Traditionally, Rohingya people had strong family bonds and provided care, especially to older members. Living in congested camps and being completely dependent on aid were our major problems. The space of each house was confined to roughly 215 square feet with three small rooms, made from bamboo, cane, tarpaulin, and waterproof plastic sheets on hilly areas. Members of 10–12 houses usually shared one latrine, which often became unsanitary due to frequent use and lack of cleanliness initiatives. Old age typically led to a decline in physical strength and mobility difficulties, making trips to the toilet stressful, especially for those who struggled with ground-level commodes. High-commode toilets were rare beside houses. The World Food Programme (WFP) tirelessly worked to ensure General Food Coverage (GFC) for all Rohingya people. A Rohingya received food worth USD 12.50 through the Family Attestation Card (FAC). A person aged 60+ years received an additional USD 3 worth of vegetables if they were the head of the family.”

### 5.9. Recognition and Discrimination

A total of 80.31% of respondents revealed that the wisdom and knowledge of an older person were beneficial for the family and community. Among them, 82.87% believed that the wisdom and knowledge of older men were more effective than women, indicating serious discrimination against older women. However, 78.38% of respondents recognized that participating in social activities was beneficial for the health and vitality of older persons. 69.70% of respondents revealed that physical exercise two to three times a week was necessary for them. The qualitative evidence from KIIs reinforces these quantitative patterns, illustrating that while intergenerational respect remains culturally embedded, practical challenges—such as crowded living spaces and food scarcity—limit older persons' autonomy. Interviewees noted that emotional neglect and lack of participation opportunities increase psychological distress, highlighting a need for inclusive social programming within the camps. KII Participant 5 raised her concerns. According to her,

“All older people liked to live with children and grandchildren. Rohingya refugees did not have opportunities to migrate outside for livelihoods. Moreover, we respected our elders from many generations. However, women were often deprived in a patriarchal society, leading to the undervaluing of their wisdom and knowledge in family and community decisions. Social needs—such as participation in activities, preservation of dignity and autonomy, and connections with family, relatives, neighbors, and friends—should be increased. Community support, especially the involvement of youth groups, was crucial in this regard.”

### 5.10. Healthcare Services

Aging and health issues were interconnected, with the highest healthcare needs in this age group. Health centers in Rohingya refugee camps provided OPD consultations, inpatient admissions, ANC services, minor and major surgeries, birth facilities, family planning services, mental health support, etc. Among respondents, 10.34% had high knowledge about health centers, including services for the older. A total of 54.91% had moderate, and 34.75% had low knowledge. Notably, 76.80% of respondents accompanied the older family member to health centers.

WHO [46] emphasized the importance of basic training for staff on core competencies in elder care, age-friendly educational materials, special queues for older persons, clean and comfortable waiting areas, and age-friendly toilets in health centers. Considering these issues, 22.03% of respondents found health centers to be good, 36.22% rated them as moderate, and 41.75% rated them as poor. Respondents identified major barriers to health services for older persons, as shown in Table 2.

In a nutshell, transportation was a difficult issue because they lived in hilly areas. Some health service-providing organizations used locally made carriers (like a comfortable chair using strong rope and bamboo carried on the shoulders of two young men) for patients. Most of the aforementioned causes could be solved. In this regard, KII Participant 7 told

“Establishing a comprehensive health service framework that included continuous monitoring and evaluation of health needs would ensure that services remained relevant and effective. Initiatives should focus on the long-term sustainability of health services, facilitating regular access to essential healthcare amenities, and fostering resilience within the community. This should include collaboration with local and international organizations to ensure equitable health services were delivered across all camps. Specific funding, programs, and partnerships focused on older refugees could ensure sustained and systematic support, addressing both immediate needs and long-term resilience-building. Additionally, empowering Rohingya community members as Community Health Workers (CHWs) to serve particularly older refugees.”

KII Participant 5 also added

“Enhancing access to age-sensitive healthcare services was essential. Programs should include periodic mental health screenings and access to specialized care via telemedicine and mobile clinics to bridge gaps in healthcare delivery. Group therapy for trauma victims, alongside individual therapy options, could provide essential support for those affected by violence and displacement. Geriatric specialists and mental health support should be made available more for older refugees.”

### 5.11. Organizational Provisions

Issues and concerns of older persons were intersectoral. All organizations had responsibilities to improve the well-being of older refugees. 48.80% of respondents agreed that existing organizational support was sufficient for older people. 35.20% had a negative response, and 16.00% were unaware. KII Participant 6 said

“Reducing vulnerability and improving resilience of older persons was a cross-cutting issue. A single organization could not address it alone. Problems of older refugees were multifaceted. They had some general problems. In addition, if he or she was disabled, living alone, or woman-headed, severe complications arose. All sectors, such as Food Security, Health, Livelihoods and Skills Development, Nutrition, Protection (Gender-Based Violence), Shelter-Camp Coordination and Camp Management (CCCM), WASH (Water, Sanitation, and Hygiene), Age and Disability, Cash & Transfers, Communication and External Relations, Emergency Preparedness and Response, Gender in Humanitarian Action, Information Management, and Assessment must mainstream prioritized issues of older refugees. The Joint Response Plan-2024 of the Rohingya Humanitarian Crisis might be a guiding document in this regard.”

In sum, respective UN and donor agencies were working in collaboration with implementing NGOs. Effective coordination and implementation were urgently needed. Initiatives should be taken to inform and raise awareness among Rohingya refugees about their interventions.

### 5.12. Age-Friendly Spaces

Several NGOs constructed age-friendly spaces (Bengali meaning: Shanti Khana) in camps for older refugees. A total of 69.52% of respondents believed that age-friendly spaces would help in the recreation of older persons. KII Participant 3 narrated

“Initially, Rohingya people were excluded from healthy recreational events. The creation of safe, age-friendly spaces within refugee camps was vital for the socialization and well-being of older refugees. They preferred it much more than gossiping on streets and tea stalls. These spaces should provide more opportunities for recreational, social, and spiritual engagement, enabling the older to interact with peers and regain a sense of community and belonging. With inadequate facilities leading to unhealthy living environments, efforts must be made to enhance these conditions to ensure a safer and more secure atmosphere.”

In summary, respondents had more acknowledgment of older men attending spaces/centers than women due to religious taboos. The most commonly reported barriers to movement in camps were steep stairs and pathways. In addition, safe mobility of older females in age-friendly spaces must be ensured.

### 5.13. Home-Based Supports and Devices

This study found that health workers visited each house in refugee camps. They provided health services to pregnant and lactating mothers, newborn infants, children, and other targeted groups. Regarding home-based support for older persons, 15.91% of respondents reported high satisfaction, 40.85% moderate, and 43.24% low. A total of 92.76% of respondents said that home-based services would be helpful for elders. It was identified that two older women had physical disabilities. They did not receive assistive devices and faced serious difficulties. Overall, 83.75% of respondents believed that counseling and assistive devices would help persons with disabilities. KII Participant 2 mentioned

“As part of community outreach activities, we visited each house, disseminating awareness messages and counseling on personal hygiene, appropriate contraceptive methods, reproductive health, sexually transmitted diseases, antenatal and postnatal care, chronic obstructive pulmonary disease, diabetes, among others. Home-based support for elder persons was comparatively lower due to resource shortages. Similarly, some persons with disabilities were excluded from receiving assistive devices.”

### 5.14. Information Dissemination

This study found that HelpAge International, Handicap International-Humanity & Inclusion (HI), and the Resource Integration Center (RIC) particularly worked in refugee camps for the well-being of older persons. The Age and Disability Working Group (ADWG) was responsible for coordination among UN agencies, donors, and implementing NGOs. 86.67% of respondents agreed that awareness building and courtyard meetings would be helpful in reducing vulnerability and improving the resilience of older people. Respondents also ranked effective ways or channels for disseminating information about health and other services, as shown in Table 3.

It was identified that functionalities of information ways/channels were scattered. For example, NGO workers arranged courtyard sessions about caring for older people, mostly to fulfill project targets. A total of 62.33% of respondents urged that home visits by health workers would be most effective in this regard. KII Participant 8 recounted

“The well-being of older people required a multi-sectoral approach involving various stakeholders such as family members, relatives/neighbors, Majis, religious leaders, youth volunteers, community leaders, Camp-in-Charge (CiC), Age and Disability Focal, implementing NGOs, medical centers, Site Management (SM), Inter-Sector Coordination Group (ISCG), and donor agencies. Strengthening the referral system was a must. Additionally, outreach efforts, including community meetings and radio broadcasts in local languages, should provide clear, accessible information on healthcare, social services, and rights protections tailored to their needs. This could help combat misconceptions about medical treatment and promote more effective utilization of healthcare resources and service provisions.”

## 6. Discussion

In this section, the findings of the present study have been critically explained to scrutinize similarity and dissimilarity with past studies. Rana et al. [56] suggest that “well-being and quality of life” is a subjective and complex concept experienced differently by individuals. While in most of the European Union, older adults prefer and tend to live alone, in many Asian countries, especially in South Asia, older people prefer to live with their families [57]. Similarly, the existing study shows that 89.92% of older persons (147 females and 192 males) live in joint families. They prefer to live with children and grandchildren, thus avoiding the anxieties of loneliness.

In Bangladesh, like most developing nations, care for older adults has traditionally been offered as unpaid care by families and relatives, backed by social, cultural, and religious norms and practices [58]. Khoda and Kröger [59] identified that although old parents are the owners of assets, they have no control over them and are often humiliated by their offspring for asking for their assets when needed. In contrast, the present study divulges that older persons are cared for by family members and relatives and are not humiliated due to a lack of land ownership or significant assets.

This study finds that the knowledge and sensitization of respondents about personal care are good, but older refugees are vulnerable due to dependency on humanitarian agencies for food, scarcity of nutritious items, inadequate and poorly managed healthcare services, fragile housing in congested camps, lack of age-friendly bathrooms and latrines, stairs in hilly areas, insufficient home-based services, limited assistive devices, weak information flows, etc. The forced displacement crisis is complex. For example, Ekoh and Walsh [60] evidenced that older migrants supported younger women who lacked male chaperones or guardians to navigate the social rules of purdah by accompanying them to resource distribution points in the Sindh Province of Pakistan. However, the current study reveals that 82.87% of respondents consider the wisdom and knowledge of older women less effective than men. Family members discourage older women from going to age-friendly spaces and engaging in physical exercise.

This community-based structure not only helps manage current refugee crises but also fosters leadership, human rights education, and project management skills that can contribute to future durable solutions. Empowering refugee communities to design and implement their projects is essential for long-term sustainable outcomes [61]. The existing study scrutinizes Rohingya community-based key actors alongside other institutions for ensuring support. Effective interventions and strategies for community engagement are urgently needed to create a supportive environment for older refugees.

Integration of quantitative and qualitative results reveals how structural and cultural factors interact to shape the well-being of older Rohingya persons. Quantitative evidence—such as the 87% acknowledgment of elders' wisdom—corresponds with qualitative insights emphasizing intergenerational respect and dependency. However, socioeconomic constraints, gender bias, and psychosocial distress frequently undermine this respect in practice. These findings echo studies by Anwar et al. [9] and Hachem et al. [13], which note that humanitarian responses often privilege material aid over emotional and social dimensions of aging. Incorporating sociocultural resilience, mutual care networks, and spiritual coping mechanisms into programming would produce a more holistic understanding of elderly well-being in displacement settings.

This study contributes new insights into the lived realities of older Rohingya refugees by examining their well-being, caregiving roles, and the importance of community engagement in humanitarian settings. While previous research has prioritized children, women, and maternal health in refugee contexts [13, 16], this study draws attention to the needs and contributions of older adults—particularly in terms of mobility, nutrition, emotional health, and access to services. These findings fill a critical gap in both academic discourse and programmatic responses.

Community engagement, often underdefined in studies on aging and displacement, is a central theme of this research. Our findings indicate that support for older persons comes not only from formal structures (such as NGOs and health workers) but also from informal sources—family members, youth volunteers, neighbors, and religious figures. These informal networks compensate for the absence of structured eldercare and help older refugees navigate daily life in the camps. This broader conceptualization of community engagement aligns with the frameworks proposed by Rass et al. [37] and Clarke [28], emphasizing the participatory role of communities in shaping humanitarian outcomes.

Compared to the existing literature on refugees, where the emphasis is often placed on women and children [13, 16], our study highlights the overlooked needs of older individuals—particularly in terms of physical health, mobility challenges, and emotional well-being. The voices of older refugees, who are often caregivers themselves or reliant on family support, bring attention to a dual role of vulnerability and resilience [62].

The gendered experiences of aging, for example, point to distinct expectations and burdens placed on older women versus men. As reported in other contexts such as Lebanon and Uganda [22, 60], older women may assume caregiving roles for grandchildren or ill relatives, often without recognition or support. Our data show that despite their caregiving roles, older women's knowledge is often undervalued, and they face barriers to participation in social spaces and physical activities. These findings echo global patterns but are shaped by specific cultural and religious contexts within the camps.

Our findings offer a unique contribution to the literature by focusing on older Rohingya refugees—a group significantly marginalized in both academic discourse and humanitarian policy. While studies from Europe often discuss aging in the context of independence and institutional care, our findings contrast sharply, revealing a strong reliance on intergenerational cohabitation and informal caregiving within the Rohingya camps. For example, in southern European countries like Italy or Spain, cultural expectations of familial support in old age remain strong [23]. These cultural comparisons emphasize the importance of context in designing age-sensitive interventions.

The expanded qualitative evidence underscores narratives of agency and resilience among older persons, rather than depicting them solely as aid recipients. Informants described elders who counsel youth, mediate disputes, and uphold religious traditions—roles aligning with HelpAge International [63] and Böcker & Hunter [21], who emphasize active social participation as protective for psychological well-being. Such testimonies highlight that resilience among older Rohingya refugees is both individual and collective, rooted in community identity and faith practices that sustain hope amid protracted uncertainty.

Moreover, the study challenges the notion that older populations are passive recipients of care. Many participants in our research were active contributors to household survival, offering spiritual guidance, domestic labor, or emotional support to younger family members. This aligns with literature that recognizes older adults as assets within displaced communities [20, 60].

One limitation of the study is its reliance on convenience sampling, which may reduce the generalizability of findings. Additionally, the absence of ethical clearance from an academic institution presents a procedural limitation, although researchers took measures to ensure informed consent and cultural sensitivity. Despite these challenges, the study fills a critical knowledge gap and sets the foundation for future work exploring how to integrate older refugees more meaningfully into humanitarian programming. Reflecting on methodology, the integration of quantitative and qualitative strands in this study demonstrates how even limited qualitative samples can generate valuable interpretive depth when analyzed alongside survey trends. As Creswell & Plano Clark [42] argue, sequential designs are particularly suitable in constrained humanitarian contexts where direct participation of vulnerable groups poses ethical risks. The combined approach thus contributes a balanced evidence base for both academic understanding and practical policy development.

Future research should build on this work by using longitudinal designs, comparing across age cohorts, and incorporating the voices of caregivers. It should also interrogate the intersections of gender, aging, and displacement in greater depth. Finally, more nuanced frameworks of community engagement—such as those proposed by Rass et al. [37] and Clarke [28]—should be applied to refugee settings, particularly with older populations in mind.

## 7. Strengths and Limitations

This study demonstrates several notable strengths. Employing a sequential mixed-methods design allowed the research to capture both statistical patterns and contextual nuances, combining data from 377 household surveys and eight KIIs across 15 Rohingya camps. This triangulated design enhanced the credibility and interpretive depth of findings by integrating the perspectives of community members, humanitarian workers, and local practitioners [54]. The study also adhered to international ethical guidelines—ensuring informed consent, confidentiality, and cultural sensitivity—through the use of gender-matched interviewers and private interview environments for vulnerable participants. These methodological and ethical safeguards contribute to the study's validity and replicability in humanitarian contexts.

Despite these strengths, several limitations should be acknowledged. The use of convenience sampling, while pragmatic in constrained and insecure field settings, restricts the representativeness of results and limits the generalizability of conclusions beyond the studied camps. The absence of formal Institutional Review Board (IRB) clearance, although mitigated by adherence to ethical protocols, indicates a degree of procedural constraint. Reliance on self-reported information introduces possible recall and social desirability bias, while the cross-sectional design prevents the examination of temporal or causal dynamics. These limitations, however, do not compromise the internal consistency of findings but highlight the context-bounded nature of field research in crisis environments.

A further methodological limitation concerns the scope of qualitative engagement, as older Rohingya individuals were not included as direct participants. The study instead relied on proxy perspectives from key informants closely involved in elderly care and humanitarian programming. While this approach safeguarded privacy and reduced ethical risk, it may not fully represent the lived experiences and agency of older refugees themselves. Future research should therefore adopt participatory qualitative methods—such as in-depth life histories, oral narratives, or focus-group discussions—to foreground older persons' voices and enhance experiential validity [60, 64]. Addressing these methodological constraints in subsequent studies will deepen understanding of resilience processes and strengthen the evidence base for age-inclusive humanitarian policy.

## 8. Recommendations

### 8.1. Improve Living Conditions for Older Refugees

Based on findings that the majority of older persons reside in overcrowded shelters and face difficulties with mobility and sanitation access, housing infrastructure must be upgraded. Age-friendly, gender-sensitive, and accessible sanitation facilities are crucial to preserve dignity and reduce health risks, especially for older women. Efforts should include providing additional nutritious food, as 79.58% of respondents recognized its importance. The WFP should expand age-specific nutritional support, particularly for older persons who are heads of households.

### 8.2. Strengthen Sustainable Community Health Systems

Given that 76.80% of respondents accompany older family members to health centers, and 41.75% rated services as poor, improvements are necessary. Establishing sustainable health services through joint funding by the RRRC, NGOs, and donors is vital. Training and employing Community Health Workers (CHWs) from the Rohingya community—as suggested by KIIs—can ensure culturally relevant care. These workers should support OPD navigation, deliver home-based care, and link older persons with essential health and social services. CHWs should also be provided with continuous education on geriatric care and psychosocial support.

### 8.3. Expand Mental Health and Psychosocial Services

Considering strong community recognition of social participation (78.38%) and mental well-being, mental health services tailored to older persons must be prioritized. Mobile clinics, group and individual therapy for trauma survivors, and mental health screenings should be integrated into healthcare delivery. Educational programs should empower older refugees to understand and access psychosocial support. Additionally, incorporating culturally sensitive practices and spiritual support could improve participation and reduce stigma.

### 8.4. Mainstream Older Persons in Humanitarian Planning

With 87.23% of respondents acknowledging the wisdom and role of older persons in families and 48.80% reporting satisfaction with existing services, humanitarian organizations must integrate the needs of older refugees into all sectoral programs. This includes WASH, nutrition, protection, livelihoods, and camp management. Budget allocations should prioritize age-specific interventions and ensure inclusivity of older persons in program design and monitoring. Advocacy campaigns should elevate the visibility of older persons and challenge against assumptions within the humanitarian response framework.

### 8.5. Expand and Promote Use of Age-Friendly Spaces

As 69.52% of respondents believed age-friendly spaces support older persons' recreation, more such spaces should be developed near shelters. Special outreach must address cultural restrictions limiting older women's participation. Safe paths and mobility infrastructure (e.g., ramps and handrails) must be built to enable access for all. These spaces should also integrate spiritual and social engagement activities, based on input from community elders and female participants.

### 8.6. Enhance Home-Based Services and Access to Assistive Devices

With only 15.91% reporting high satisfaction with home-based care and many older persons lacking assistive devices, home visit programs should be scaled up. These should include personal care, medication delivery, and psychological support. Priority must be given to older persons with disabilities—ensuring assistive tools such as wheelchairs, hearing aids, or high-commode seats are distributed based on need. Programs should encourage intergenerational caregiving within families to combat isolation and promote mutual support across age groups.

### 8.7. Improve Information Dissemination and Referral Systems

Given the fragmented awareness channels and the fact that 86.67% supported courtyard meetings and awareness activities, a coordinated communication strategy is needed. CHWs and Majis should lead household-level outreach with support from NGOs. A functional, transparent referral system must be established for older persons, ensuring timely access to services. Accessible formats (visuals, audio, language translations) should be used to inform older populations. Monitoring mechanisms should evaluate how well older people understand and use the services introduced.

### 8.8. Promote Multisectoral Collaboration and Advocacy

All stakeholders—including UN agencies, implementing NGOs, camp authorities, and the ADWG—must work collaboratively to address the cross-cutting challenges faced by older refugees. The Joint Response Plan (JRP) should include measurable objectives and outcome indicators specific to the well-being of older persons. Shared accountability frameworks, regular inter-agency reviews, and community participation should be integrated into implementation plans.

## 9. Conclusion

Older Rohingya refugees in Bangladesh face multifaceted challenges, including inadequate age-sensitive infrastructure, limited healthcare, and poor access to nutritious food and support services. Despite strong familial caregiving traditions, their well-being remains compromised by systemic gaps in humanitarian coordination and service delivery. This study offers critical insights into the lived experiences of older refugees, highlighting their roles as both dependents and informal caregivers. It calls for age-inclusive, community-driven interventions that acknowledge the resilience and contributions of older persons. Strengthening interorganizational collaboration and tailoring services to the needs of aging populations are essential steps toward an inclusive humanitarian response.

## Figures and Tables

**Table 1:** KII participants.

Respondent	Sex	Age	Education	Marital status	Organization/designation	Experience in year	Living place
1	Male	28	MSc	Married	Teacher	7	Camp 18
2	Female	25	Grade-7	Married	Health worker	5	Camp 22
3	Male	50	Grade-9	Married	Maji	—	Camp 11
4	Male	46	Grade-8	Married	Religious leader	—	Camp 24
5	Female	21	Grade-8	Married	Case worker	3	Camp 14
6	Male	49	Masters	Married	Additional RRRC	7	Cox's Bazar
7	Male	32	Masters	Married	HelpAge	4	Cox's Bazar
8	Female	35	LLM	Married	UN Agency	6	Cox's Bazar

*Note:* Source: Authors.

**Table 2:** Causes of health service problems (multiple answers).

Causes	Frequency	Rank
Waiting time (i.e., time-consuming in health centers)	252	1
Transportation problem	233	2
Delay in getting an appointment with a doctor	197	3
Noncooperation of service staff	121	4
No separate unit for older	111	5
Lack of accompanying	94	6
Lack of age-friendly toilet	61	7
High cost	38	8

**Table 3:** Effective way/channel for information dissemination (multiple answers).

Way/channel	Frequency	Rank
Home visit of health workers	235	1
Volunteers	197	2
Majis (headmen)	194	3
Family/relatives/neighbors	158	4
Camp-in-charges (CiCs)	122	5
Community leaders	77	6
Protection desks	66	7
Women and girls' safe spaces	47	8
Medical facilities	44	9
Suggestion boxes	4	10
Hotlines	3	11

## Data Availability

The data that support the findings of this study are available from the corresponding author upon reasonable request.
